# Antioxidant capacities and in vitro anti-microbial activities of rice (*Oryza sativa var* Bajong) from Borneo

**DOI:** 10.1186/s13065-025-01453-x

**Published:** 2025-04-09

**Authors:** Xiu Qian Lam, Heng Yen Khong, Siow Phing Tay, Isabel Lim Fong

**Affiliations:** 1 Department of Paraclinical Sciences, Faculty of Medicine and Health Sciences, Universiti Malaysia Sarawak, 94300 Kota Samarahan, Sarawak Malaysia; 2 Faculty of Applied Sciences, Universiti Teknologi MARA Sarawak Branch, 94300 Kota Samarahan, Sarawak Malaysia

**Keywords:** *Oryza sativa var* Bajong, Amino acids, Phenolics, Flavonoids, Antioxidant, Antimicrobial

## Abstract

Rice contains antioxidants and phenolic components that exert anti-inflammatory and anticancer properties. Different geographical areas produce rice with various chemical constituents and phytochemicals, in turn these confer differential protective effects including antimicrobial and anticancer properties. Sarawak rice, *Oryza sativa var* Bajong (Bajong), a fragrant dark purple rice grain harvested from two locations in Sarawak, namely interior Lubok Nibong (LN) and coastal Sri Aman (SA), was assessed for their antioxidant properties and antimicrobial activities. The rice was extracted using methanol solvent and evaluated for total phenolic content (TPC), total flavonoid contents (TFC), as well as their antioxidant and antimicrobial activities based on the Folin–Ciocalteu assay, the aluminium calorimetric method, 2,2-diphenyl-1-picrylhydrazyl (DPPH) free radical scavenging activity and disk diffusion assay, respectively. Using dual-wavelength measurement, Bajong LN showed 2.16% higher amylose content than Bajong SA. Furthermore, 24-h extract of Bajong LN and 48-h extract of Bajong SA exhibited high antioxidant properties (34–70 µg/mL) and were rich in phenolic (46.54 ± 2.62 mg GAE/g; 25.28 ± 3.91 mg GAE/g) and flavonoid contents (5.53 ± 0.36 mg QCE/g; 7.7 ± 2.19 mg QCE/g) respectively. It is interesting to note that 72-h Bajong extract exhibited the largest zone of inhibition (9–9.3 mm) against Gram-negative *Pseudomonas aeruginosa* (*Ps. aeruginosa*), which correlated to the high TPC and TFC despite a reduction of antioxidant activity due to prolonged extraction hours. These significant results conferred added value to a staple, health-promoting Bajong had warranted it to be further investigated as a nutraceutical and pharmaceutical crop.

**Supplementary Information:**

The online version contains supplementary material available at 10.1186/s13065-025-01453-x.

## Introduction

Rice (*Oryza sativa* L.) is an important staple food that is consumed by approximately half of the world’s population [14] with most Asians prefer white rice to pigmented rice. Over the years, studies had revealed the extensive nutritional and medicinal properties of pigmented rice, namely from bran, germ, and endosperm, respectively [5, 6, 23, 55]. There is a scarcity of study on unpolished, pigmented rice grain as routinely consumed.

In different geographical areas, different variants of rice contain different ratio of chemical constituents. Therefore, their chemoprotective and chemopreventive properties differ among the selected rice types. Several factors that dictate these properties include type of land use, soil, geology, slope, vegetation, aspect, and climate control, as these affect the quantity and quality of surface water that irrigates the paddy field [58]. Furthermore, Esmail & Rajab [12] reported that the chemical composition of irrigation waters with same electrical conductivity increased the magnesium/calcium and sodium/calcium ratio in soil composition, which benefitted the plant.

Pigmented rice contains phenolics and flavonoids as secondary metabolites that have anticarcinogenic, antimutagenic, antibacterial, antifungal, anti-inflammatory, immune system promoting, cholesterol-lowering, anti-diabetic effects, and antioxidant activities [5, 6, 18, 52, 69]. These chemoprotective effects have different mechanisms and pathways, including cyclooxygenase pathway, free-radical scavenging pathway, mitogen-activated protein kinase pathway, and inflammatory cytokines signalling pathway [18, 52, 69]. In Malaysia, Sarawak is blessed with various colourful, fragrant rice varieties. Many of these have yet to be chemically characterised and analysed, including their antioxidant, chemoprotective and chemopreventive potentials. Among these rice varieties, *Oryza sativa* var Bajong (Bajong), which belongs to one of the darkest varieties (dark purple), has shown to be a more robust crop in the presence of physiological parameters. Its intense colour and stability are influenced by pH, light, structure, and temperature [15].

This study aims to determine the amylose content, total phenolic and total flavonoid contents, antioxidant potential and antimicrobial activities of Bajong crude extracts from two locations designated for the planting of Bajong, namely Sri Aman and Lubok Nibong in Sarawak. The outcomes of this study conferred added value including nutraceutical and pharmaceutical values that warranted further investigation as promising chemopreventive and chemoprotective agents.

## Materials and methods

### Collection and preparation of rice samples

A total of 5 kg high-quality, freshly harvested Bajong rice were collected from the farmers in Sri Aman (SA) and Lubok Nibong (LN) in Sarawak, Malaysia. The collected rice samples were stored in vacuum-sealed bags before further processing.

### Extraction of rice samples

Bajong SA and Bajong LN were ground into fine powder respectively. A batch of 20 g fine powdered rice samples were extracted in 200 mL 80% methanol in conical flasks (Thermo Fisher Scientific, Australia). The plant material to solvent ratio 1:10 at room temperature for 24, 48, and 72 hours (h), respectively. To minimize the degradation of phenolics and flavonoids, the conical flasks were parafilm and covered with a box. After extraction, each of these extracts was filtered through Whatman™ No.1 filter paper (Whatman™, England) and concentrated *in vacuo* using a rotary evaporator R-200 (BUCHI, Flawil, Switzerland). The crude was freeze-dried by Lyovapor L-200 Pro (BUCHI, Flawil, Switzerland), sealed in an airtight container and stored at 4 ^◦^C prior to further experiments. The weights and yield percentages (%w/w) of these crude extracts were calculated using the equation below:

$$\text{Percent yield }\left(\text{\%}\right)=\frac{Actual\,Yield}{Theoretical\,Yield} \times 100\%$$

### Determination of amylose content

In this study, the dual-wavelength method was chosen due to its high precision and accuracy [29, 71]. Soxhlet extraction was performed to extract bioactive compounds, especially fat and oil from rice before quantifying amylose, as lipids and bioactive compounds may interfere with the quantification of amylose by forming complexes with amylose. Soxhlet extraction offers better extraction efficiency for bioactive compounds and lipids compared to maceration, as maceration using methanol has time and yield trade-offs [8].

The Bajong samples were prepared using Soxhlet extraction method with 2 g of finely powdered Bajong rice in 80% methanol at 40–45 °C for 16 hrs. The amylose contents (% w/w) of rice samples were determined using the amylose and amylopectin as standards. The established calibration curve (absorbance versus the amylose content) showed a linear range of amylose concentrations between 0 and100% (Figure S1 and Figure S2). This was used to determine the amylose content in the rice samples. Triplicates were performed and analysed.

### Total phenolic content

The total phenolic content (TPC) of the rice extracts was assessed with the Folin– Ciocalteu assay as described by [22]. The control blank was prepared using methanol while gallic acid (Sigma-Aldrich, Australia) served as the positive control.  The calibration curve was plotted, showing a linear range of concentrations between 0–500 µg/ mL (Figure S3) in the presence of Folin– Ciocalteu reagent (Sigma-Aldrich, Australia). The TPC of rice extracts was calculated as mg of gallic acid equivalent (GAE)/g dry weight (dw), using the equation obtained from the standard calibration curve as means ± standard deviation (SD) of triplicates.

### Total flavonoid content

The total flavonoid content (TFC) of rice extracts was determined using the aluminium calorimetric method described by Phung et al. [51] with slight modifications. Quercetin served as the standard reference, and its calibration curve was established using a two-fold serial dilution starting from 50 to 0.39 µg/ mL (Figure S4). The negative control was prepared using the same procedures but using methanol. The TFC of rice extracts can be calculated as mg of quercetin equivalents (QCE)/g dw, using the equation obtained from the standard calibration curve as means ± SD of triplicates.

### 2-diphenyl-1-picrylhydrazyl (DPPH) radical scavenging activity

The antioxidant activity of the Bajong extracts was determined using 2,2-diphenyl-1-picrylhydrazyl (DPPH) free radical scavenging activity assay described by [47] with slight modifications with Quercetin (Sigma-Aldrich, Australia) as the positive control. Quercetin is a flavonoid with potent free radical scavenging because it reacts efficiently with DPPH to ensure reproducible results. This high reactivity makes it an excellent standard to evaluate free radical scavenging activity [49].

The mixture was swirled gently, and the absorbance was measured after 30 min of incubation in the dark at room temperature. The negative control was the 95% ethanol and measured immediately at 0 min. The absorbance of the mixture was measured at 517 nm using a UV–vis spectrometer. The DPPH free radical scavenging activity was calculated as follows:

$${\text{DPPH (\% ) = Free radical scavenging activity (\% ) = [(Absorbance control - Absorbance sample)/Absorbance control]}} \times {\text{100}}$$

The sample concentration required for the half-maximal inhibitory concentration (IC_50_) values was calculated by plotting the percent inhibition of DPPH radicals (%) against the sample concentrations. IC_50_ values were reported as means ± SD of triplicates. A higher value of DPPH inhibition (lower IC_50_) represents a more potent antioxidant activity.

### Antimicrobial analysis

To have a qualitative assessment or a visual representation of inhibition zones, the Bajong extracts were tested for antimicrobial activity using the disk diffusion method (Kirby-Bauer method). A total of 9 clinically important bacteria, encompassing Gram-positive and Gram-negative species were purchased from American Type Culture Collection (ATCC), USA. The Gram-positive bacteria were *Staphylococcus aureus* (**ATCC No. 25923**), *Staphylococcus epidermidis* (**ATCC No. 12228**), *Streptococcus pyogenes* (**ATCC No. 19615**) while Gram-negative bacteria were *Pseudomonas aeruginosa* (**ATCC No. 109246**), *Klebsiella pneumonia* (**ATCC No. BAA-1705**), *Escherichia coli* (**ATCC No. 1100101**), *Shigella* species (**ATCC No. 23354**), *Salmonella typhimurium* (**ATCC No. 14028**) and *Proteus mirabilis* (**ATCC No. 29906**).

This disk diffusion method was performed as described by Vora et al. [63] with slight modifications. Bacterial lawns for the 9 bacteria were prepared on Mueller–Hinton agar (Oxoid, UK). Rice extracts were prepared at a concentration of 1 mg/ mL. Sterile empty disks (Milipore, USA) were placed on the lawn and 20 uL of each rice extract in Mueller–Hinton broth (MHB) (Oxoid, UK) were pipetted onto the sterile disks. MHB was vehicle control while positive control Levofloxacin 5 µg disk (Oxoid, Basingstoke, UK) was placed for Gram-negative bacteria species, while positive control Vancomycin 30 µg disk (Oxoid, UK) was used for Gram-positive bacteria. These agar plates were incubated at 37 °C incubator and observed at 24, 48, and 72 h. The zone of inhibition (ZOI) diameter of all extracts and positive controls were measured and recorded in mm. The experiment was performed and analysed in triplicates.

### Statistical analysis

All data were analysed using SPSS 26.0 for Windows 10 (SPSS Inc., Chicago, IL, USA). A paired-T test was conducted on different geographical regions of Bajong. The same test was also performed to evaluate the relationship between the TPC and TFC of Bajong. There was also a *post hoc* test analysis to assess Bajong chemical constituents’ association with different extraction times. A p-value ≤ 0.05 denoted statistical significance.

## Results

### Weights and percentages yield of rice extracts

From the starting material of 20 g finely powdered rice, the weights and yield percentages (% w/w) of the Bajong SA extracts were 0.1697 g (0.85%), 0.2166 g (1.08%), 0.4080 g (2.04%) for 24-, 48- and 72-h extractions, respectively. Similarly, the weights and yield percentages (%w/w) of the Bajong LN extracts were 0.2523 g (1.26%), 0.2770 g (1.39%), 0.2985 g (1.49%) for 24-, 48-, and 72-h extractions, respectively (Table S1). The results showed that the increase in duration of extraction corresponded with the increase amount and yield percentage of the extract. Additionally, Bajong LN yielded higher than Bajong SA at 24- and 48-h extraction duration. Contrarily, at 72-h extraction duration, the yield percentage of Bajong SA was higher than that of Bajong LN.

### Determination of Amylose Content

The amylose content of Bajong was determined by single- (620 nm) and dual-wavelength (620/510 nm) spectrophotometry, showing higher amylose content in Bajong LN (4.65%, 5.47%) than Bajong SA (2.57%, 3.31%) respectively **(**Table 1**)**.

**Table 1:** Single and dual-wavelength amylose content determination of Bajong

Bajong Extracts	Amylose (%) at single wavelength (620 nm)	Amylose (%) at dual wavelength (620 nm / 510 nm)
LN	4.65	5.47
SA	2.57	3.31

There are five classifications of amylose content: waxy (0–2%), very low (2–10%), low (10–20%), intermediate (20–25%), high (25–33%) (Juliano 1992)

### Total phenolic content (TPC)

In this study, the TPC of 24-, 48-, and 72-h Bajong LN extracts were found to be 46.54 ± 2.62 mg, 42.12 ± 2.67 mg, and 52.17 ± 3.29 mg GAE/g extract respectively while those of 24-, 48-, and 72-h Bajong SA extracts recorded lower TPC of 9.61 ± 2.01 mg, 25.28 ± 3.91 mg, and 25.06 ± 1.83 mg GAE/g extract, respectively (Table 2).

**Table 2:** TPC, TFC and Antioxidant capacities of Bajong extracts from two different geographical regions

Extraction time	Rice sample						Positive control
	Rice LN			Rice SA			
	24	48	72	24	48	72	
TPC (mg GAE/g extract ± SD)	46.54 ± 2.62	42.12 ± 2.67	52.17 ± 3.29	9.61 ± 2.01	25.28 ± 3.91	25.06 ± 1.83	–
TFC (mg QCE/g extract ± SD)	5.53 ± 0.36	4.68 ± 0.37	10.56 ± 0.73	2.72 ± 0.31	7.7 ± 2.19	9.12 ± 0.94	–
DPPH IC_50_ (µg/mL)	34.84	37.69	38.1	128.62	69.17	158.96	2.3

*TPC* total phenolic content, *GAE* gallic acid equivalents, *TFC* total flavonoid content, *QCE*, quercetin equivalents, *DPPH IC*_*50*_ DPPH scavenging activity, *SD* standard deviation

The 72-h Bajong LN extract exhibited the highest TPC value compared to those of 24- and 48-h extracts. The TPC (LN) decreased in 48-h and the subsequent increase in 72-h during extraction reflects the interplay between adsorption onto the matrix and desorption back into the solvent [4]. Interestingly, the 48- and 72-h Bajong SA extracts both recorded similar higher TPC values than that of 24-h extract (Table 2).

The phytochemical content variations of Bajong are influenced by soil characteristics and environmental conditions, which affect stress responses, plant metabolism, and nutrient absorption. The complex interactions between soil moisture, nutrients, pH, climate, altitude, light exposure, and stress factors can influence the metabolic pathways of Bajong, resulting in variations in the production of phytochemicals between Bajong SA and LN [43, 48]. During the field work and interview sessions with the farmers, it was found that the farmers in SA were issued with Agricultural Research Centre (ARC) fertilisers and strictly followed the instruction given while those in LN were not issued with these fertilisers. This may result in less response to stress in Bajong SA than Bajong LN, which was further evidenced by the lighter colour of Bajong SA than Bajong LN [39]. This concurred with the findings that TPC of Bajong SA extracts were drastically lower TPC than those of Bajong LN. This marked difference could be because the two areas where the Bajong was planted have different soil types, nutrient availability and climate conditions, that could affect phenolic synthesis [27, 34].

To determine the relationship between the TPC content and the duration of extraction of 24-, 48-, and 72-h, the results showed longer duration corresponded significantly to higher TPC, especially those of Bajong LN were significant in 48- and 72-h extracts (p-value = 0.005). While TPC values of Bajong SA were significant at 24- and 48-h (p-value < 0.001), as well as 24- and 72-h (p-value < 0.001) extraction times respectively. There were also significant differences present by comparing the TPC content of Bajong (LN and SA) at 24-h (p-value = 0.001), 48-h (p-value = 0.004), and 72-h (p-value = 0.009) extraction time respectively (Table 3**; **Fig. 4).

**Table 3:** The significant difference in TPC and TFC values for different geographical regions of Bajong Rice

Paired-T test	Paired Samples	Mean	SD	P-value	Post Hoc Test	Samples		Extraction time	P-value
Pair 1	TPCLN24-TPCSA24	36.93	2.19	0.001*	Post Hoc 1	TPCLN		24–48	0.109
Pair 2	TPCLN48-TPCSA48	16.84	1.84	0.004*				24–72	0.054
Pair 3	TPCLN72-TPCSA72	27.11	4.4	0.009*				48–72	0.005*
Pair 4	TFCLN24-TFCSA24	2.81	0.08	< 0.001**	Post Hoc 2	TPCSA		24–48	< 0.001**
Pair 5	TFCLN48-TFCSA48	-3.03	1.85	0.106				24–72	< 0.001**
Pair 6	TFCLN72-TFCSA72	1.44	1.58	0.255				48–72	0.924
Pair 7	TPCLN24-TFCLN24	41.01	2.97	0.002*	Post Hoc 3	TFCLN		24–48	0.089
Pair 8	TPCLN48-TFCLN48	37.45	2.88	0.002*				24–72	< 0.001**
Pair 9	TPCLN72-TFCLN72	41.6	3.42	0.002*				48–72	< 0.001**
Pair 10	TPCSA24-TFCSA24	6.89	2.28	0.034*	Post Hoc 4	TFCSA		24–48	0.005*
Pair 11	TPCSA48-TFCSA48	17.57	5.34	0.029*				24–72	0.001*
Pair 12	TPCSA72-TFCSA72	15.93	0.91	0.001*				48–72	0.257

*SD*, standard deviation, *TPC* total phenolic content, *TFC* total flavonoid content, *LN* Bajong (Lubok Nibong), *SA* Bajong (Sri Aman), 24, 24-h-extraction-time; 48, 48-h-extraction-time; 72, 72-h-extraction-time; * p-value < 0.01; ** p-value < 0.001

### Total flavonoid content (TFC)

Flavonoids are the most abundant and well-studied phytochemicals with antioxidant and biochemical properties efficacious against various diseases. The TFC of Bajong was quantified in terms of quercetin using a standard calibration curve equation (R^2^ = 0.9986). For rice harvested in LN, it had a total TFC of (5.53 ± 0.36 mg QCE/g extract), (4.68 ± 0.37 mg QCE/g extract) and (10.56 ± 0.73 mg QCE /g extract) respectively, for 24, 48, and 72 h of extraction time (Table 2). The TFC (LN) decreased at 48-h and the subsequent increase at 72-h during extraction. This can be influenced by similar factors such as matrix composition that has adsorption onto the matrix and desorption from the matrix [65]. It can also be affected by the chemical properties of flavonoids, solvent properties and the extraction methods.

In comparison, rice harvested in SA had TFC values of (2.72 ± 0.31 mg QCE /g extract), (7.7 ± 2.19 mg QCE /g extract) and (9.12 ± 0.94 mg QCE /g extract) respectively for 24, 48, and 72 h of extraction time (Table 2). It was shown that Bajong extracted in 72 extraction hours from both places (LN; SA) had the highest TFC values (10.56 ± 0.73 mg QCE /g extract; 9.12 ± 0.94 mg QCE /g extract) respectively compared to 24 h and 48 h extraction period.

To determine the relationship between the TFC content of Bajong at 24-, 48-, and 72-h extraction time, TFC values of Bajong LN were found significant at 24 to 72 h (p-value < 0.001) and 48 to 72 h (p-value < 0.001). While TFC values of Bajong SA were significant at 24 to 48 h (p-value = 0.005) and 24 to 72 h (p-value = 0.001) with different extraction times. There were also substantial differences present between the TFC content of different geographical areas (LN and SA) at 24 h (p-value < 0.001) extraction time. Paired-T tests were also conducted by comparing the TPC and TFC of Bajong. The TPC and TFC values of Bajong LN 24 h, 48 h and 72 h of extraction time have a p-value of 0.002. The TPC and TFC values of Bajong SA only have a p-value of 0.001 at 72 h of extraction time **(**Table 3**; **Fig. 4**)**.

### DPPH radical scavenging activity

**Fig. 1:**
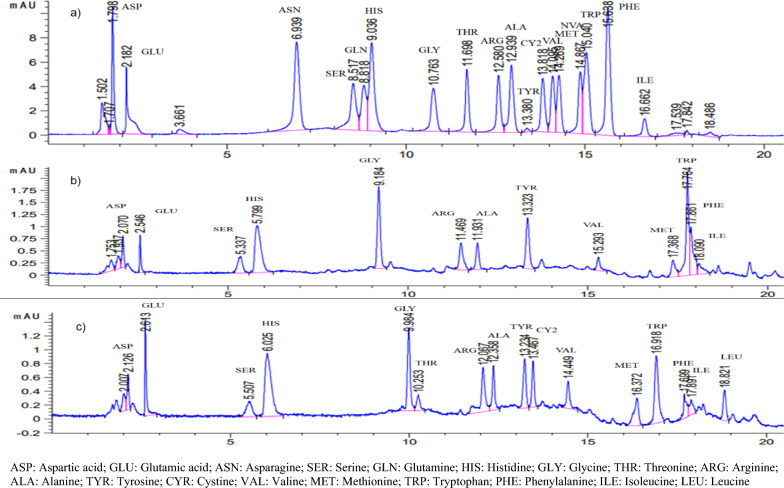
Amino acid profile of: **a** Standards mixture; **b** Bajong Rice (Lubok Nibong); **c** Bajong Rice (Sri Aman)

**Fig. 2:**
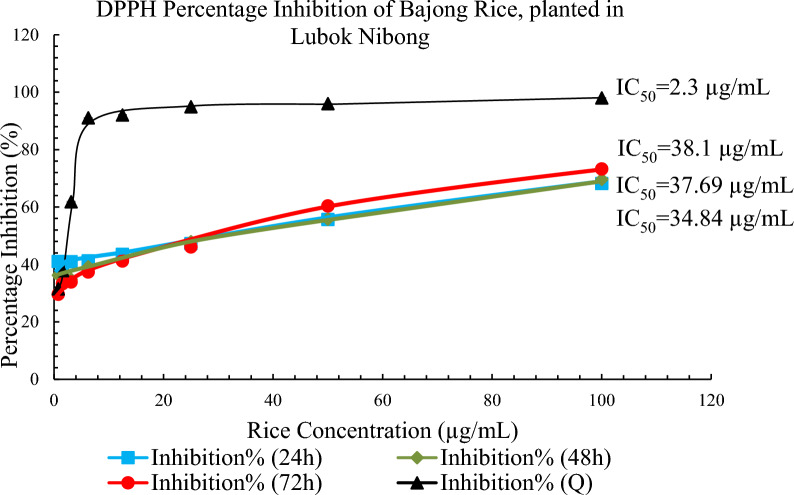
Antioxidant activity of Bajong planted in LN

Plants contain a variety of antioxidants that resist environmental threats. In this study, the DPPH radical-scavenging effect of Bajong extracts was observed in a concentration-dependent manner up to the given concentration (100 µg/mL). As shown in Table 2, Figs. 1 and 2, the DPPH inhibition activity of Bajong (LN and SA) extracts of varying concentrations, along with Quercetin IC_50_ of 2.3 µg/mL was obtained. The percentage inhibition of Bajong LN extracted after 24 h showed the highest antioxidant activity, followed by Bajong extracted after 48 and 72 h. Based on DPPH percentage inhibition activity, Bajong LN extracted after 24 h was more potent in scavenging DPPH and exhibited more potent antioxidant activity with IC_50_ of 34.84 µg/mL than Bajong LN extracted after 48 h and 72 h, with IC_50_ of 37.69 µg/mL and IC_50_ of 38.1 µg/mL, respectively.

The percentage inhibition of scavenging activities of the Bajong SA extracted after 48 h showed the highest total antioxidant capacity, followed by Bajong extracted after 24 and 72 h respectively. Based on the DPPH radical scavenging activity, Bajong SA extracted after 48 h showed a strong scavenging free radical activity with IC_50_ of 69.17 µg/mL. Bajong SA extracted after 24 h incubation period had percentage inhibition with IC_50_ of 128.62 µg/mL, and Bajong SA extracted after 72 h with IC_50_ of 158.96 µg/mL. These showed that the IC_50_ value increased from 48 h, 24 h, and 72 h, accordingly, in which Bajong SA extracted after 48 h showed the most potent potential in scavenging free radical activity.

It was found that the potency of Bajong LN extract was higher after 24 h than 48 h and 72 h, whereas the TPC and TFC were higher at 72 h. As antioxidant activity is not solely determined by phenolic and flavonoid compounds, where other compounds such as vitamins, amino acids and carotenoids can be extracted by methanol or ethanol and these possess strong antioxidant properties [13, 50, 68]. Thus, the antioxidant potency can be high, with lower TPC and TFC at 24 h. Similarly, Bajong SA showed higher potency with higher TPC at 48-h, but lower TFC at 24 h. Other factors such as synergistic effects, assay-specific mechanism, or undetected compounds can cause the disproportional to the antioxidant potency in TPC and TFC.

### Antimicrobial activity

A disk diffusion assay was conducted on Bajong extracts (LN and SA) to assess their antimicrobial activities at working stock of 1 mg/mL. The results from Table 4 showed that both rice extracts showed inhibition zones on *Ps. aeruginosa* in the disk diffusion assay. When Bajong extract (LN) was applied on Gram-negative *Ps. aeruginosa*, the zone of inhibition (ZOI) diameter was 8.5 mm, 8.8 mm, 8.5 mm (24-h extraction); 9 mm, 9 mm, 8.5 mm (48-h extraction); 9 mm, 9 mm, 9 mm (72-h extraction). At the same time, the ZOI diameter for Bajong extract (SA) was 8 mm, 8 mm, 9 mm (24 h extraction period); 9 mm, 9.3 mm, 9 mm (48 h extraction period); 9 mm, 9 mm, 9.3 mm (72 h extraction period) respectively. These demonstrated that the ZOI of Bajong extracts (LN and SA) increased when the exposure duration to Bajong extract was longer. No antimicrobial activities were observed in the other Gram-positive (*Staph. aureus*, *Staph. epidermidis,* and *Strep. pyogenes)* or Gram-negative bacteria (*Kleb. pneumonia*, *E. coli*, *Shigella* species, *Salmonella typhimurium* and *Proteus mirabilis)*.

**Table 4:** Antimicrobial potential of Bajongmethanol extractswith different extraction and at  different exposure durations

Rice Samples	Bajong LN									Bajong SA									Control		
Extraction duration (h)	24			48			72			24			48			72			-		
Exposure duration (h)	24	48	72	24	48	72	24	48	72	24	48	72	24	48	72	24	48	72	24	48	72
Gram-positive bacteria																			Vancomycin (30 µg)		
*Staph. aureus*	Χ	Χ	Χ	Χ	Χ	Χ	Χ	Χ	Χ	Χ	Χ	Χ	Χ	Χ	Χ	Χ	Χ	Χ	22	21.5	21
*Staph. epidermidis*	Χ	Χ	Χ	Χ	Χ	Χ	Χ	Χ	Χ	Χ	Χ	Χ	Χ	Χ	Χ	Χ	Χ	Χ	21	21	21
*Strep. pyogenes*	Χ	Χ	Χ	Χ	Χ	Χ	Χ	Χ	Χ	Χ	Χ	Χ	Χ	Χ	Χ	Χ	Χ	Χ	20	21	21
Gram-negative bacteria																			Levofloxacin (5 µg)		
*Ps. aeruginosa*	8.5	8.8	8.5	9	9	9	9	9	9	8	8	9	9	9.3	9	9	9	9.3	38	40.5	42
*Kleb. pneumonia*	Χ	Χ	Χ	Χ	Χ	Χ	Χ	Χ	Χ	Χ	Χ	Χ	Χ	Χ	Χ	Χ	Χ	Χ	31	31	31
*E. coli*	Χ	Χ	Χ	Χ	Χ	Χ	Χ	Χ	Χ	Χ	Χ	Χ	Χ	Χ	Χ	Χ	Χ	Χ	41	41	40
*Shigella sp.*	Χ	Χ	Χ	Χ	Χ	Χ	Χ	Χ	Χ	Χ	Χ	Χ	Χ	Χ	Χ	Χ	Χ	Χ	37	39	38
*S. typhimurium*	Χ	Χ	Χ	Χ	Χ	Χ	Χ	Χ	Χ	Χ	Χ	Χ	Χ	Χ	Χ	Χ	Χ	Χ	38	39	40
*P. mirabilis*	Χ	Χ	Χ	Χ	Χ	Χ	Χ	Χ	Χ	Χ	Χ	Χ	Χ	Χ	Χ	Χ	Χ	Χ	43	44	41

The zone of inhibition (ZOI) (mm) when different areas of Bajong were treated on Gram-positive and Gram-negative bacteria on Mueller–Hinton Agar. ‘X’ defines no inhibition occurs

## Discussion

The exclusive use of methanol as an extraction solvent can extract more phenolic content, as supported by Ghasemzadeh et al. (2018) [16] and Altemimi et al. [1]. This is because methanol is a polar solvent that can extract polar and moderately polar compounds, such as phenolics, flavonoids, and certain alkaloids [3]. Hence, in this study, 80% methanol was used to increase the extraction recovery further, as water enhances the solubility of certain phenolic compounds and provides a higher yield [11].  In this study, Bajong LN has a higher yield during 24-and 48- h extraction than Bajong SA. This may be due to several factors such as plant traits, environmental factors, soil fertility and the composition of the rice from two different areas. Conversely, at 72-h, Bajong SA showed better yield than Bajong LN.  Our study found that Bajong LN (single, dual: 4.65%, 5.47%) has a higher amylose content than the Bajong SA (2.57%, 3.31%) (Table 1). The amylose content obtained from Bajong (LN and SA) is considered low (0–6%) (Juliano 1992), which indicates that Bajong contains a high level of amylopectin, which may be retrograded during the hydrolysis to make the Bajong less sensitive to digestion [61]. It is also supported by Jang & Xu [25] where purple rice contains high dietary fibre in the germ and bran fractions and caused a delay in starch granules’ accessibility to digestive enzymes in the digestive system. Hence, this inferred that when Bajong is consumed, a slow starch digestion can reduce the risk of metabolic syndrome amd type 2 diabetes [19]. A study by Li et al. [38] also demonstrated that high amylose corn had a better antioxidant activity compared to the typical and waxy genotypes as observed in these Bajong extracts, especially  Bajong LN with a low glycaemic index (GI) of starches and low amylopectin. Bajong with a low GI is highly beneficial in individuals with metabolic disorders, as it promotes better insulin sensitivity, blood sugar regulation, lipid profile improvement, weight management, inflammation reduction and gut health. Meanwhile, unpolished Bajong tends to have the lowest GI compared to their polished forms [26, 32]. All the above finsingsmake Bajong  ideal for dietary interventions in metabolic health.

Additionally, Bajong LN exhibited more TPC and TFC than those of Bajong SA (Table 2). This is evidenced by the darker colour of Bajong LN compared to Bajong SA, in which darker pigments reveal higher TPC and TFC contents [30]. The data also shows that Bajong from LN (IC_50_: 34–39 µg/mL) is more potent than Bajong from SA (IC_50_: 69–159 µg/mL). This result was supported by Wen [67], in which Bajong LN was found to contain higher antioxidant capacity than  the other rice varieties such as Bali, Pandan, Wangi Mamut, MR219, Bubuk, Biris and Bario in Sarawak. It suggested that Bajong LN has higher antioxidant potential to transfer hydrogen atoms to free radicals by homolytic O–H bond cleavage via free radical scavenging mechanism. With higher antioxidant potential, Bajong has greater strength to inactivate ROS production enzymes and activate antioxidant enzyme systems to repair and remove ROS-induced damage [20, 56, 60].

**Fig. 3:**
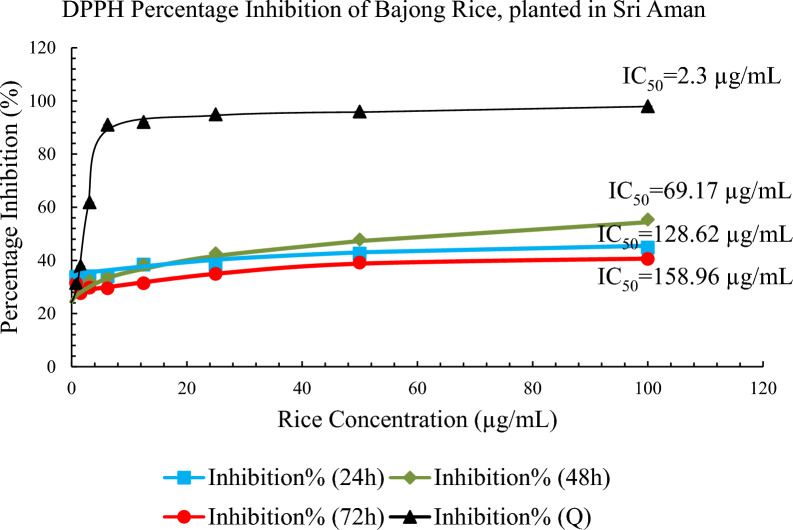
Antioxidant activity of Bajong planted in SA

**Fig. 4:**
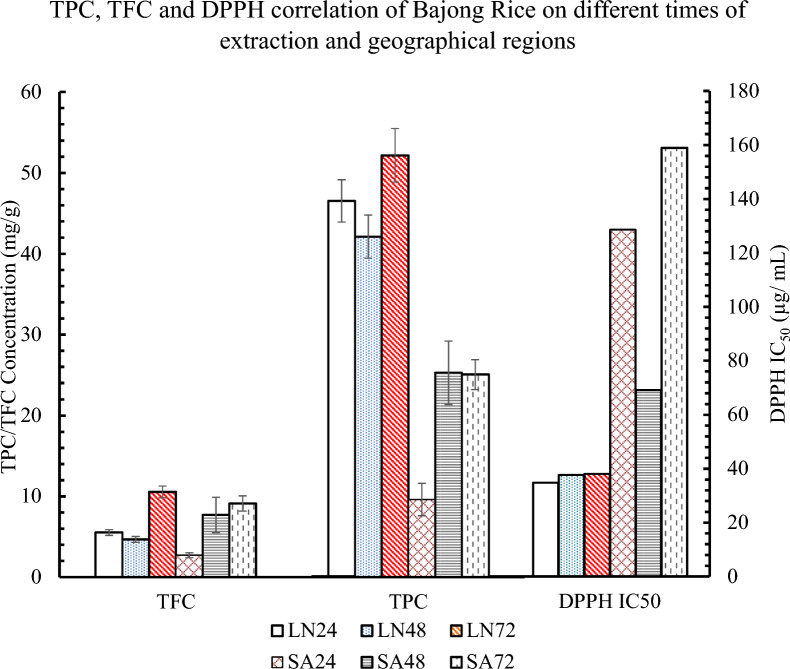
Correlation of TPC, TFC values, and DPPH free radicals scavenging activity. Note: *TPC* total phenolic content, *TFC* total flavonoid content; DPPH IC_50_ DPPH scavenging activity; *LN* Bajong (Lubok Nibong), *SA* Bajong (Sri Aman); 24, 24-h-extraction-time; 48, 48-h-extraction-time; 72, 72-h-extraction-time

**Fig. 5:**
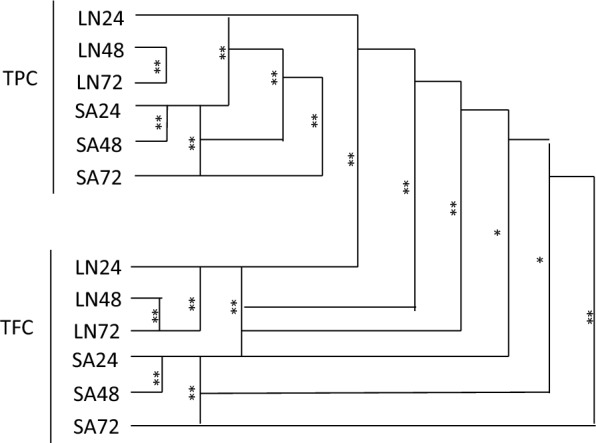
The significant difference of TPC and TFC values of *Oryza sativa var* Bajong. *TPC* total phenolic content, *TFC* total flavonoid content, *LN* Bajong (Lubok Nibong), *SA* Bajong (Sri Aman); 24, 24-h-extraction-time; 48, 48-h-extraction-time; 72, 72-h-extraction-time; *p-value < 0.01; **p-value < 0.001

However, the percentage inhibition of Bajong LN decreased at 72h  extraction, with higher TPC and TFC values but low-potency antioxidants (Fig. 3). This result agrees with [16] study, where the highest phytochemical content and antioxidant activity were observed in black rice (darker colour) followed by red or brown rice. The antioxidant capacities depend on the chemical structure feature of the compounds (variation in hydrophobicity and hydrogen donating ability) and the chemical matrix in which it is located. [45]. The number and position of phenolic hydroxyls (hydrogen donating groups), methoxy, and carboxylic groups (electron donating groups) are directly related to the antioxidant capacities [31]. With fewer hydroxyl groups present, the antioxidant capacity of Bajong is reduced. As such, high TPC and TFC values do not necessarily correspond higher antioxidant property (Figs. 4 and 5).

Interestingly, the percentage inhibition of Bajong SA increased gradually from 24 to 48 h and decreased in 72 h. The sudden drop in the antioxidant activity at 72 h of the extraction period can be due to the extended exposure to oxygen and light, where oxidation and chemical losses of the bioactive compounds happen [17]. Therefore, the results show that the antioxidant capacity of Bajong could be affected by several issues reported above. These antioxidant capacities are not able to correlate directly to ROS. Therefore, an antimicrobial assay was performed to verify the antimicrobial activity of a compound regardless of their antioxidant potential.

In this study, both Bajong extracts (LN and SA) that had undergone 72 h of extraction were efficacious against Gram-negative *Ps. aeruginosa* at 72 h of exposure duration. High TPC and TFC compounds are known to   target specific pathways or DNA replication enzymes of the bacteria [57]. This result is supported by a study that the most newly developed antibiotics are active against Gram-positive bacteria due to the impermeable outer membrane found in Gram-negative bacteria. [21]. This conferred a valuable antimicrobial potential to Bajong to exhibit antimicrobial activity against Gram-negative *P.aeruginosa.* There are several factors that may govern this ability, including the ability to disrupt the oxidative stress, efflux pump, and quorum sensing pathways. One of the mechanisms that increase bacterial susceptibility to Bajong in *Ps. aeruginosa* is efflux pump blockage. While *Ps. aeruginosa* is found to have  strong efflux pumps, including MexXY-OprM, MexAB-OprM MexEF-OprN, which actively export antibiotics and other antimicrobial compounds [42, 46], but Bajong contains certain flavonoids, such as kaempferol and quercetin, which can bind to the efflux pump proteins, interfering with their function and preventing the release of antimicrobial substances [64]. These flavonoids can disrupt proton flux, reducing the ATP hydrolysis and proton gradients for the pump operation. Phenolics and flavonoids can also downregulate efflux pump genes, reducing the expression, causing *P. aeruginosa* to be more susceptible to antimicrobials [59].

*Ps. aeruginosa* had been found to exhibit resistance to many common antibiotics due to its lipopolysaccharide-rich outer membrane [53]. Certain phenolics and flavonoids can bind to these lipopolysaccharides and destabilize the outer membrane, thereby increasing permeability. Pseudomonas aeruginosa exhibits resistance to many common antibiotics due to its lipopolysaccharide-rich outer membrane. Certain phenolic and flavonoid compounds can bind to these lipopolysaccharides, destabilizing the outer membrane and increasing its permeability. This alteration facilitates the entry of antimicrobial agents, including antibiotics. Additionally, the inner phospholipid bilayer may also be disrupted. In view of this, phenolics and flavonoids derived from Bajong  may form pores in the membrane of* Ps. aeruginosa leading to ion leakage.* A *Ps. aeruginosa* and ultimately contributes to cell collapse [41]. The synergistic effects of polyphenols are less pronounced in Gram-negative bacteria due to their complex outer membrane structure, and  are very specific to the phenolics present [54]. With the purported membrane-disruption and efflux pump inhibition abilities to impair the bacterial defences, these make  Bajong a promising alternative for further study in antimicrobial therapy.

From Table 2, the TPC value of Bajong SA at 48 h of extraction time is similar to the TPC value at 72 h of extraction time. It can be concluded that the TPC is fully extracted at 48 h extraction time, and a longer extraction duration  is unnecessary to extract more phenolics. The data indicates that Bajong extracted for 48h is more superior  than those of 24- and 72- extraction h, as it recorded  higher antioxidant activity (low IC_50_). This study  also showed that Bajong LN has higher TPC, TFC, and subsequently greater potency than Bajong SA in terms of mopping up free radicals.

In addition, TFC of Bajong (LN and SA) extracted for 24 h correlated with each other unlike those underwent 48- and 72-h extraction. At longer extraction duration, marked differences were observed in the TFC of Bajong from these two interior and constal areas despite being  same rice variety.

## Conclusion

The findings from this study revealed that Bajong exhibits health-promoting effects with high amino acids, tryptophan, glycine, total phenolic and flavonoid content and potent antimicrobial properties. Moreover, Bajong LN has higher tryptophan, glycine, amylose, phenolics, flavonoids, and antioxidant capacities than Bajong SA. This is in agreement with Tyagi et al. [62] that reported the most significant amount of phenolics, flavonoids, and antioxidant capacities were found in purple rice, which can be considered functional food and nutraceutical components for illness prevention. Hence, Bajong can be regarded as a functional food and nutraceutical component that promotes health benefits and illness prevention. Therefore, it is worth to explore further its wide  potential applications in the pharmaceutical and medical sectors. Additionally, its nutrient-rich composition, high antioxidant potential, low cost, and easy availability have many advantages for commercialization potential.

## Recommendation

To significantly improve the bioactive properties of Bajong that has high amylopectin, various processing methods like the enzymatic treatments, high-pressure processing, fermentation, and encapsulation can be administered [3, 7, 28, 40, 66]. This is because  partial gelatinization of amylopectin helps improve digestibility and releases bound bioactive compounds such as polyphenols [10, 37]. Amylopectin hydrolyzation into smaller oligosaccharides and sugars can also have prebiotic properties [9, 33]. Therefore, Bajong with high amylopectin content can be transformed into potent nutraceutical products for commercial use.

It is challenging for the antioxidant and antimicrobial properties observed in vitro being replicated in in vivo, particularly in human clinical studies, yet it can be possible when accounting for factors like bioavailability, metabolism, synergistic interaction and delivery methods. Several studies have shown that human trials have successfully demonstrated these in specific cases [24, 44], but advanced formulations, long-term studies, and personalized approaches could improve consistent outcomes.

## Supplementary Information

Supplementary Material 1.

## Data Availability

Data is provided within the manuscript or supplementary information files.
