# Supplementary material for: Antioxidant capacities and in vitro anti-microbial activities of rice (Oryza sativa var Bajong) from Borneo
Source: BMC Chem. 2025 Apr 9;19(1):92. doi: 10.1186/s13065-025-01453-x (PMC11983904; doi:10.1186/s13065-025-01453-x)
Supplement: Supplementary file 1 — Supplementary Material 1. [file 13065_2025_1453_MOESM1_ESM.docx]

**Supplementary materials**

**Figure S1**: Standard calibration curve of amylose content at a single wavelength (620nm).

y = 0.0031x -0.0281

R^2^ = 0.9992

**Figure S2**: Standard calibration curve of amylose content at dual wavelength (620/ 510 nm)

**Figure S3**: **Standard calibration curve of gallic acid.**

**Figure S4**: **Standard calibration curve of Quercetin.**

**Table S1**: Weights and yield percentages (%w/w) of the rice crude extracts.

| Extraction time | Rice from SA | |  | Rice from LN | |
| --- | --- | --- | --- | --- | --- |
|  | Weight (g) | Yield (%) |  | Weight (g) | Yield (%) |
| 24 hours | 0.1697 | 0.8485 |  | 0.2523 | 1.2615 |
| 48 hours | 0.2166 | 1.083 |  | 0.277 | 1.385 |
| 72 hours | 0.408 | 2.04 |  | 0.2985 | 1.4925 |

*Raw rice sample weight = 20.0 g

Ramata-Stunda, A., Petriņa, Z., Valkovska, V., Borodušķis, M., Gibnere, L., Gurkovska, E., & Nikolajeva, V. (2022). Synergistic Effect of Polyphenol-Rich Complex of Plant and Green Propolis Extracts with Antibiotics against Respiratory Infections Causing Bacteria. *Antibiotics, 11*(2), 160. <https://doi.org/10.3390/antibiotics11020160>.
